# Suburethral Adjustment With 8/4 Calibration Versus Conventional Technique in Transobturator Tape (TOT) Placement: A Comparative Clinical Study

**DOI:** 10.7759/cureus.99039

**Published:** 2025-12-12

**Authors:** Juan Carlos Aguilar Ortega, María Esther Suárez Garcia, Andres Rivera, Christopher Kaleb Romero Ríos, Lorenzo E Aragón Conrado

**Affiliations:** 1 Obstetrics and Gynecology, Hospital Militar Escuela "Dr. Alejandro Dávila Bolaños", Managua, NIC; 2 School of Medicine, Hospital Militar Escuela "Dr. Alejandro Dávila Bolaños", Managua, NIC; 3 Medical Education, Escuela de Medicina Teniente Coronel y Doctor Sergio Martinez Ordoñez, Managua, NIC; 4 Medical Education, Hospital Militar Escuela "Dr. Alejandro Dávila Bolaños", Managua, NIC

**Keywords:** quality of life, stress urinary incontinence, suburethral, surgical mesh, urethropexy

## Abstract

Objective

To compare the postoperative clinical outcomes of suburethral adjustment with 8/4 calibration versus the conventional technique in the placement of transobturator suburethral tape (TOT) for female stress urinary incontinence.

Materials and methods

A prospective, longitudinal, and analytical cohort study was conducted on 60 women diagnosed with stress or mixed urinary incontinence with a predominance of stress, treated at the "Dr. Alejandro Dávila Bolaños" Military School Hospital in Managua, Nicaragua. Participants were assigned by convenience sampling to two groups: 40 women received the conventional surgical technique and 20 were treated with the 8/4 adjustment technique. Demographic characteristics, surgical procedure duration, length of hospital stay, presence of complications, and quality of life, the latter measured by the ICIQ-SF questionnaire, were evaluated.

Results

The average age was similar in both groups (~54 years). The surgical procedure duration was significantly shorter in the group treated with the 8/4 technique (23 min 47 sec vs. 27 min 30 sec; p=0.03, independent samples t-test). No significant differences were observed in the length of hospital stay or the incidence of immediate complications (at 24 hours and 10 days post-surgery) between the groups (p > 0.05, Chi-square test). At three months, the conventional technique group had a higher incidence of complications (22.5% vs. 5%; p=0.02, independent t-test). Quality of life significantly improved in both groups after surgery (p < 0.01, paired t-test), with no significant differences in the degree of improvement between them (p=0.45, independent t-test).

Conclusion

The 8/4 adjustment technique proved to be a safe and effective option for the treatment of stress urinary incontinence, associated with a lower incidence of medium-term postoperative complications, without compromising the improvement in quality of life compared to the conventional technique. These findings suggest that the 8/4 adjustment represents an advantageous alternative that could be considered in clinical practice.

## Introduction

Urinary incontinence (UI) is defined as the involuntary loss of urine through the urethra [1,2]. It is a common problem in the female population, with a prevalence that increases with age [3-5]. It is estimated that between 15% and 50% of women aged 30 to 60 years experience some degree of UI. In addition to its physical repercussions, this condition has a considerable psychological and social impact, negatively affecting patients' quality of life and generating a high demand for resources in health services [6-8].

Initial treatment includes behavioral therapies and lifestyle modifications. However, in many cases, a definitive surgical approach is required, which has evolved toward less invasive, more effective techniques with lower morbidity, aiming to minimize medium- and long-term postoperative complications [9].

The risk of developing stress urinary incontinence is associated with well-established factors such as aging, obesity, multiparity, a history of hysterectomy, the presence of comorbidities, the use of diuretics, and participation in high-impact physical activities. Increased exposure to these factors has contributed to the occurrence of urinary incontinence in young women [10, 11].

At the Department of Gynecology and Obstetrics of the Hospital Militar Escuela “Dr. Alejandro Dávila Bolaños” (HMEADB), the placement of a transobturator suburethral sling (TOT) is routinely performed using the Mayo scissors adjustment technique, which has shown optimal results in the immediate postoperative period. However, this technique has limitations regarding standardization, as it largely depends on the surgeon's clinical judgment and experience. In contrast, the 8/4 adjustment technique provides a more objective method, as it is based on quantifiable measurements using Hegar dilators, an 8 mm dilator inside the urethra and a 4 mm dilator between the urethra and the sling, thereby reducing intraoperative variability, facilitating reproducibility, and allowing for standardized tensioning. This approach has been associated with consistent sling placement and improved predictability of postoperative outcomes (e.g., reduced risk of urethral obstruction).

Therefore, the aim of this study was to compare the postoperative clinical outcomes between both techniques and determine whether the 8/4 adjustment provides similar or superior benefits in terms of complications and quality of life.

## Materials and methods

Study design

An observational, analytical, prospective, and longitudinal cohort study was conducted in the Department of Gynecology and Obstetrics at the Hospital Militar Escuela "Dr. Alejandro Dávila Bolaños" (HMEADB) in Managua, Nicaragua.

Population and sample

The study population consisted of 60 women diagnosed with stress urinary incontinence (SUI) who underwent surgery between January and June 2024. A non-probabilistic convenience sampling method was used based on surgical volume. Patients were assigned to two groups: 40 women were treated using the conventional technique, and 20 were treated using the 8/4 adjustment technique. Participant selection was conducted through a review of physical and electronic clinical records.

Inclusion and exclusion criteria

The study included patients diagnosed with SUI or mixed urinary incontinence with a stress predominance who underwent surgery at HMEADB and completed follow-up at three evaluation points: 24 hours, 10 days, and three months post-surgery. Exclusion criteria included incomplete follow-up, deficient clinical records, prolonged hospital stay for reasons unrelated to the procedure, a history of prior suburethral tape placement, or recurrence of SUI.

Variables and clinical assessment

Sociodemographic variables (age, origin, marital status, occupation, ethnicity) and gynecological-obstetric history (parity, body mass index, menopausal status, and hormone therapy use) were evaluated. Clinical characteristics included the presence of associated pelvic floor dysfunctions and the severity of incontinence.

Severity of incontinence

Baseline severity was assessed during the preoperative consultation using the Sandvik Severity Index (Incontinence Severity Index) [12]. This validated tool calculates a severity score by multiplying the reported frequency of leakage (four-level scale) by the estimated amount of urine lost (three-level scale). The resulting score ranges from 0 to 12, categorizing incontinence as mild (1-2), moderate (3-6), severe (7-9), or very severe (10-12).

Quality of life

Quality of life was assessed using the International Consultation on Incontinence Questionnaire - Short Form (ICIQ-SF) [13]. This validated tool evaluates three key domains: frequency of urinary leakage, perceived quantity of leakage, and overall interference with everyday life. The total score is the sum of these items, ranging from 0 to 21, where higher scores indicate greater symptom severity and a worse impact on quality of life. The questionnaire was self-administered by patients preoperatively and at the three-month follow-up.

Surgical procedure

All patients underwent transobturator suburethral tape (TOT) placement.

Conventional Technique

The mesh was adjusted using Mayo scissors as a reference, leaving an approximate separation of 3 to 5 mm from the urethra.

8/4 Calibration Technique

An 8-mm Hegar dilator was placed intraurethrally, and a 4-mm Hegar dilator was positioned between the mesh and the urethra to ensure a precise 4-mm suburethral space, as originally described by Ludwig et al [14].

Patients presenting with concomitant pelvic organ prolapse underwent additional indicated surgical procedures (e.g., vaginal hysterectomy, anterior/posterior colpoplasty, or perineoplasty) during the same surgical session. However, to isolate the impact of the calibration technique, operative time was defined strictly as the duration of the TOT placement. This interval was measured from the initial vaginal incision for the suburethral sling to the closure of the vaginal and inguinal incisions, excluding the time required for any concomitant gynecological procedures.

Data collection and follow-up

Data were collected during preoperative consultations, hospitalization, and follow-up visits using a Google Forms® electronic data sheet validated by a pilot test. Patients were evaluated in person at 24 hours, 10 days, and 3 months post-surgery. At each follow-up, postoperative complications were recorded, and the ICIQ-SF questionnaire was readministered at the three-month mark.

Statistical analysis

Data were analyzed using SPSS software version 27 (IBM Corp., Armonk, NY, USA). Quantitative variables were expressed as means and standard deviations, while qualitative variables were reported as absolute and relative frequencies. The Chi-square test was used to evaluate associations between categorical variables. Means were compared using the Student's t-test or Welch's t-test based on the homogeneity of variances (Levene's test). Intra-group comparisons (pre- vs. postoperative ICIQ-SF scores) were analyzed using a dependent samples t-test. Normality was assumed based on the central limit theorem.

Ethical considerations

The study was approved by the institutional and university ethics committees and was conducted in accordance with the ethical principles of the Declaration of Helsinki. Data confidentiality was strictly guaranteed for academic purposes.

## Results

Sociodemographic, personal, and gynecological-obstetric characteristics

A total of 60 patients were analyzed: 20 underwent the suburethral adjustment with 8/4 calibration technique and 40 underwent the conventional technique. In the 8/4 group, 50.0% were between 51 and 60 years old, while in the conventional group, the 61 to 70 age range predominated (32.5%). No statistically significant differences were found in the age distribution between both groups (χ² = 6.64; p = 0.249), indicating demographic homogeneity. The predominant marital status was "married" in both groups: 65.0% in the 8/4 group and 62.5% in the conventional group (χ² = 0.667; p = 0.716). Most patients came from urban areas, with no statistically significant differences between the groups.

Personal and gynecological-obstetric histories were similar: average number of pregnancies (3.5 vs. 3.2), deliveries (2.4 vs. 2.35), and cesarean sections (0.35 vs. 0.38). The proportion of postmenopausal women was also comparable (70.0% in the 8/4 group vs. 65.0% in the conventional group). The average body mass index was 31.9 kg/m² in the 8/4 group and 31.0 kg/m² in the conventional group. The use of hormone replacement therapy was low and without significant differences.

Severity of stress urinary incontinence and pelvic floor dysfunctions

The severity of urinary incontinence had an overall mean Sandvik index score of 5.82 points with a predominance of moderate grades (scores between 4 and 8). The most frequent pelvic floor disorder was anterior compartment defect grade II+ (29.6% in the 8/4 group and 24.4% in the conventional group). The prevalence of urogenital atrophy was significantly higher in the conventional group (34.1% vs. 7.4%; p < 0.05). Other dysfunctions were also identified, such as involvement of the apical and posterior compartments, cervical elongation, and fecal incontinence, with differences in their distribution between the groups.

Additional gynecological surgery included vaginal hysterectomy, anterior and posterior colpoplasty, and perineoplasty. Vaginal hysterectomy was more frequent in the 8/4 group (36.0%), while anterior colpoplasty predominated in the conventional group (46.7%). The average surgical time was significantly shorter in the 8/4 group (23.78 ± 9.77 minutes) compared to the conventional group (27.50 ± 19.52 minutes; p < 0.05) (Table 1).

**Table 1:** Duration of the procedure This table presents the operative time for transobturator tape (TOT) placement using two techniques: the 8/4 calibration technique and the conventional technique. Data are reported as mean procedure duration, standard deviation (SD), first quartile (Q1), and third quartile (Q3) in hours, minutes, and seconds.

Duration of the procedure (minutes)	8/4 Technique	Conventional technique
Mean	00:23:47.90	00:27:30.07
Standard Deviation	00:09:45.64	00:19:30.81
Q1	00:16:00.00	00:16:30.00
Q3	00:30:00.00	00:30:00.50

At three months, 95.0% of the patients in the 8/4 group remained free of complications; only 5.0% developed de novo urgency. In the conventional group, 73.8% remained without complications; however, 26.3% presented complications distributed as follows: 14.3% presented de novo urgency, 7.2% required surgical reintervention for infravesical obstruction or mesh extrusion, and 2.4% presented a granuloma or exposure of the TOT sling.

Postoperative Evolution in Both Groups

All patients achieved spontaneous voiding without the need for re-catheterization. No complications were reported in the first 24 postoperative hours, allowing for a hospital stay of one day or less. At ten days post-surgery, 94.7% of the patients in the 8/4 group had no complications; 5.3% reported mild pain. In the conventional technique group, 87.5% were free of complications, 10.0% experienced postoperative pain, and 2.5% developed a urinary tract infection (Table 2).

**Table 2:** Complications at 10 days. The table allows direct comparison of each complication type between techniques. Percentages were calculated based on the total number of patients in each group.

Complications at 10 Days	8/4 Technique		Conventional Technique	
	Frequency	%	Frequency	%
None	18	94.7%	35	87.5%
Postoperative pain	1	5.3%	4	10.0%
Urinary tract infection	0	0.0%	1	2.5%
Hemorrhage	0	0.0%	0	0.0%
Bladder injury	0	0.0%	0	0.0%
Urinary retention	0	0.0%	0	0.0%
Surgical site infection	0	0.0%	0	0.0%

The Pearson correlation test applied to the relationship between surgical technique and the presence of complications at 10 days yielded a coefficient of 0.327 (p = 0.160), indicating no statistically significant association. Levene's test showed homogeneity of variances (p = 0.280). The independent samples t-test also showed no significant differences in the mean of complications (t = 0.529; df = 58; p = 0.599), with a mean difference of 0.05 (95% CI: -0.1390 to 0.2391) (Table 3).

**Table 3:** Student's t-test for Equality of Means for Complications at 10 Days This table presents the results of a Student’s t-test comparing the mean frequency of complications at 10 days post-transobturator tape (TOT) placement between the 8/4 calibration technique and the conventional technique. The table reports the t-value, degrees of freedom (df), two-tailed p-value, mean difference, standard error of the difference, and the 95% confidence interval (CI) of the difference.

Variable	t	df	p-value (2-tailed)	Mean Difference	Std. Error Difference	95% Confidence Interval of the Difference (Lower)	95% Confidence Interval of the Difference (Upper)
Complications 10 days post-op	0.529	58	0.599	0.050	0.09446	-0.1390	0.23908

At three months, 95.0% of the patients in the 8/4 group remained free of complications; only 5.0% developed de novo urgency. In the conventional group, 73.8% remained without complications; however, 26.3% presented complications distributed as follows: 14.3% presented de novo urgency, 7.2% required surgical reintervention for infravesical obstruction or mesh extrusion, and 2.4% presented a granuloma or exposure of the TOT sling (Table 4).

**Table 4:** Complications at 3 months Complications are compared between the 8/4 calibration technique and the conventional technique, including no complications, urinary urgency, surgical reintervention, granuloma formation, material exposure, mesh extrusion, and chronic pain. The 8/4 group shows a higher proportion of patients without complications compared to the conventional group.

Complications at 3 Months	8/4 Technique		Conventional Technique	
	Frequency	%	Frequency	%
None	19	95.0%	31	73.8%
De novo urinary urgency	1	5.0%	6	14.3%
Surgical reintervention	0	0.0%	3	7.2%
TOT sling granuloma	0	0.0%	1	2.4%
Material exposure	0	0.0%	1	2.4%
Mesh extrusion	0	0.0%	0	0.0%
Chronic pain	0	0.0%	0	0.0%

The Pearson correlation between the surgical technique and complications at three months was -0.124 (p = 0.604), again indicating no significant correlation. However, Levene's test showed heterogeneity of variances (p = 0.0002) at the expense of the conventional group, so Welch's t-test was used, which revealed a statistically significant difference (t = -14.46; df = 56; p = 8.42 × 10⁻²⁰), with a mean difference of 0.225 (95% CI: 0.02294 to 0.42706) (Table 5). These findings support that the 8/4 technique is associated with a more favorable medium-term postoperative evolution, with a lower incidence and variability of complications compared to the conventional technique.

**Table 5:** Welch's t-test (unequal variances) for Complications at Three Months Data include t-value, degrees of freedom (df), two-tailed p-value, mean difference, standard error of the difference, and 95% confidence interval. The results indicate a statistically significant difference in complications between the two techniques at three months postoperatively.

Variable	t	df	p-value (2-tailed)	Mean Difference	Std. Error Difference	95% Confidence Interval (Lower)	95% Confidence Interval (Upper)
Complications 3 months post-op	-14.46	56	8.42 × 10⁻²⁰	0.22500	0.10088	0.02294	0.42706

Quality of Life

Quality of life, measured by the ICIQ-SF questionnaire, significantly improved in both groups at three months post-intervention. The mean preoperative score was 10.9 in the 8/4 group and 8.3 in the conventional group. At three months, most patients obtained scores close to zero, indicating substantial improvement or resolution of urinary symptoms. No statistically significant differences were found between the two groups regarding the magnitude of improvement (p = 0.079) (Table 6).

**Table 6:** Comparison of Preoperative, Postoperative, and Change in ICIQ-SF Scores Between the 8/4 Technique and the Conventional Technique ICIQ-SF scores are shown for preoperative, three-month postoperative assessments, and the change in scores after TOT placement. Data include mean, standard deviation (SD), quartiles (Q1, median, Q3), minimum, and maximum, allowing direct comparison between the 8/4 calibration technique and the conventional technique.

ICIQ Scores	Conventional Technique	8/4 Technique
Preoperative	Values	Values
Mean	8.3	10.9
SD	3.3	4.2
Q1 (25th percentile)	5.0	7.0
Median	8.0	10.5
Q3 (75th percentile)	10.0	14.0
Min	4.0	5.0
Max	17.0	20.0
Postoperative (3 months)		
Mean	0.13	0.00
SD	0.79	0.00
Q1	0.00	0.00
Median	0.00	0.00
Q3	0.00	0.00
Min	0.00	0.00
Max	5.00	0.00
Change in ICIQ-SF	Values	Values
Mean	8.25	10.78
SD	3.27	4.07
Min	4.00	5.00
Max	17.00	20.00

## Discussion

The high proportion of patients from urban areas and those identified as married also reflects the social determinants influencing access to specialized gynecological care [12,13].

The predominance of postmenopausal women in the sample supports evidence linking estrogen deficiency to degeneration of pelvic floor support structures [15]. In this context, the low use of hormone replacement therapy, as observed in this and other studies [16], suggests an opportunity to strengthen preventive strategies in women at high risk of pelvic floor weakness.

Overweight and obesity, present in most patients, are recognized risk factors for SUI and its postoperative recurrence [17]. Therefore, they should be considered key and modifiable elements in the comprehensive management of these patients.

Regarding the surgical technique, the inclusion of additional procedures such as colpoplasty, hysterectomy, or perineoplasty showed no direct relationship with the type of TOT used. However, their frequency and type could influence the postoperative course. Performing multiple interventions during a single surgical act may increase complexity, prolong recovery, and affect functional outcomes [18-21]. In the 8/4 group, vaginal hysterectomy was performed more frequently, while anterior colpoplasty was more common in the conventional group, both representing approaches that may have differential effects on pelvic statics [15].

In terms of anatomical defects, anterior compartment II+ was the most prevalent, consistent with studies identifying it as the one most associated with SUI [11]. On the other hand, urogenital atrophy was significantly more prevalent in the conventional group, which may be related to both hormonal factors and surgical effects. Previous literature has suggested a possible association between surgical manipulation and the development of postoperative atrophy, an area that warrants further research [13,18].

The immediate postoperative outcomes were favorable in both groups, with complete functional recovery within the first 24 hours, no need for recatheterization, and no relevant complications. This safety profile is consistent with existing literature describing TOT as a minimally invasive procedure with rapid recovery [18]. At 10 days, the observed complications were mild and comparable between groups, reinforcing its short-term safety, as also reported in other multicenter studies [20,21].

However, at three months, significant differences were identified: the group undergoing the 8/4 technique had a lower rate of late complications, particularly de novo urinary urgency. This complication, widely documented in sling techniques, has an incidence ranging from 10% to 20%, depending on the technique used and the patient's anatomical characteristics [22]. The lower incidence observed in the 8/4 group could be related to a more precise and individualized suburethral adjustment. This finding warrants further investigation in controlled clinical trials.

Analysis of the ICIQ-SF questionnaire showed a significant improvement in pre- and postoperative scores in both groups, supporting the clinical effectiveness of both techniques. Although a non-probabilistic convenience sample was used, the random assignment of patients to each group helped balance baseline characteristics and reduce potential bias.

The higher preoperative score observed in the 8/4 group may be explained by random baseline imbalance rather than a directed selection of more severe cases. This initial difference did not affect the comparative postoperative efficacy. Previous studies have also reported similar improvements in SUI symptoms following TOT procedures [22,23].

Furthermore, a significant correlation was identified between initial scores and the magnitude of improvement, suggesting that patients with more severe baseline symptoms could achieve greater benefits from the procedure. The lower variability observed in the postoperative scores of the 8/4 group also suggests a more homogeneous response, further supporting the potential value of this technique as a standardized therapeutic option.

Although the differences in ICIQ-SF score reduction between techniques did not reach statistical significance, the favorable trend toward greater improvement with the 8/4 technique justifies its evaluation in studies with larger sample sizes. These preliminary results suggest that the 8/4 technique may offer clinical advantages in the surgical treatment of SUI without compromising effectiveness.

The interpretation of our results should be considered in light of several limitations. First, the study utilized a non-probabilistic convenience sampling design with a relatively small sample size (N=60). The allocation of patients resulted in an uneven distribution between groups (2:1 ratio), which was determined by the surgical case flow and technique preference during the study period rather than by randomization. Additionally, an a priori power calculation was not performed, which may limit the statistical power to detect differences in rare adverse events. Second, the follow-up period was limited to three months; therefore, long-term outcomes regarding efficacy, recurrence, and late complications remain to be evaluated. Finally, as a single-center study, the generalizability of these findings to other clinical settings may be limited. Despite these constraints, we believe this study provides valuable preliminary evidence regarding the benefits of standardizing the TOT technique.

## Conclusions

The results of this study indicate that both transobturator cystourethropexy techniques are effective for the treatment of stress urinary incontinence, offering favorable immediate postoperative recovery. However, the 8/4 calibration technique demonstrated a significantly shorter operative time and a lower rate of medium-term complications, specifically de novo urinary urgency. These findings suggest that the calibrated 8/4 suburethral adjustment is a promising surgical alternative that may reduce procedural variability and optimize functional outcomes particularly in patients with higher baseline severity without compromising overall effectiveness. Nevertheless, given the study's design limitations, these advantages should be considered preliminary and require confirmation through larger, randomized controlled trials with long-term follow-up.
