# Correction: Suburethral Adjustment With 8/4 Calibration Versus Conventional Technique in Transobturator Tape (TOT) Placement: A Comparative Clinical Study

**DOI:** 10.7759/cureus.c396

**Published:** 2026-01-26

**Authors:** Juan Carlos Aguilar Ortega, María Esther Suárez Garcia, Andres Rivera, Christopher Kaleb Romero Ríos, Lorenzo E Aragón Conrado

**Affiliations:** 1 Obstetrics and Gynecology, Hospital Militar Escuela "Dr. Alejandro Dávila Bolaños", Managua, NIC; 2 School of Medicine, Hospital Militar Escuela "Dr. Alejandro Dávila Bolaños", Managua, NIC; 3 Medical Education, Escuela de Medicina Teniente Coronel y Doctor Sergio Martinez Ordoñez, Managua, NIC; 4 Medical Education, Hospital Militar Escuela "Dr. Alejandro Dávila Bolaños", Managua, NIC

This article has been corrected to add a citation to the following sentence found in the Materials and Methods section:

"An 8-mm Hegar dilator was placed intraurethrally, and a 4-mm Hegar dilator was positioned between the mesh and the urethra to ensure a precise 4-mm suburethral space, as originally described by Ludwig et al [14]."

All subsequent in-text citations have been renumbered accordingly.

